# Acute Stroke Care and Thrombolytic Therapy Use in a Tertiary Care Center in Lebanon

**DOI:** 10.1155/2014/438737

**Published:** 2014-07-16

**Authors:** Mazen J. El Sayed, Tharwat El Zahran, Hani Tamim

**Affiliations:** ^1^Department of Emergency Medicine, American University of Beirut Medical Center, P.O. Box-11-0236, Riad El Solh, Beirut 1107 2020, Lebanon; ^2^Emergency Medical Services and Prehospital Care Program, American University of Beirut Medical Center, P.O. Box-11-0236, Riad El Solh, Beirut 1107 2020, Lebanon; ^3^Department of Internal Medicine, American University of Beirut Medical Center, P.O. Box-11-0236, Riad El Solh, Beirut 1107 2020, Lebanon

## Abstract

*Background*. Thrombolytic therapy (rt-PA) is approved for ischemic stroke presenting within 4.5 hours of symptoms onset. The rate of utilization of rt-PA is not well described in developing countries. *Objectives*. Our study examined patient characteristics and outcomes in addition to barriers to rt-PA utilization in a tertiary care center in Beirut, Lebanon. *Methods*. A retrospective chart review of all adult patients admitted to the emergency department during a one-year period (June 1st, 2009, to June 1st, 2010) with a final discharge diagnosis of ischemic stroke was completed. Descriptive analysis was done followed by a comparison of two groups (IV rt-PA and no IV rt-PA). *Results*. During the study period, 87 patients met the inclusion criteria and thus were included in the study. The mean age was found to be 71.9 years (SD = 11.8). Most patients arrived by private transport (85.1%). Weakness and loss of speech were the most common presenting signs (56.3%). Thirty-three patients (37.9%) presented within 4.5 hours of symptom onset. Nine patients (10.3%, 95% CI (5.5–18.5)) received rt-PA. The two groups (rt-PA versus non rt-PA) had similar outcomes (mortality, symptomatic intracerebral hemorrhage, modified Rankin scale scores, and residual deficit at hospital discharge). *Conclusion*. In our setting, rt-PA utilization was higher than expected. Delayed presentation was the main barrier to rt-PA administration. Public education regarding stroke is needed to decrease time from symptoms onset to ED presentation and potentially improve outcomes further.

## 1. Introduction

Stroke is the leading cause of disability among adults in the United States (US) and in Europe. Nearly 800,000 new strokes occur per year in the US and 1.1 million in Europe [1, 2]. Stroke burden is even higher in developing low and middle-income countries where the average age of patients with stroke is 15 years younger than that in high-income countries [3].

Ischemic strokes (caused by in situ thrombosis, embolism, or systemic hypoperfusion) are the most common type of strokes worldwide (87%) [1]. Restoring perfusion of the ischemic brain region through recanalization of the occluded artery is the immediate goal of acute stroke treatment [4].

Alteplase (recombinant tissue plasminogen activator or rt-PA), the only approved medical therapy for acute ischemic stroke, has been shown to be safe and effective if given within 3 hours of symptoms onset [5]. Recent guidelines have recommended extending the treatment window with intravenous (IV) rt-PA up to 4.5 hours from symptoms onset based on results from the European Cooperative Acute Stroke Study (ECASS) III trial [6]. There is no benefit beyond 4.5 hours, with the possible advantage perhaps offset by risk (symptomatic intracerebral hemorrhage (SICH) and death) [7].

The utilization of thrombolytic therapy for acute stroke patients in the developing countries is low (less than 1% to 3%) compared to developed countries (up to 10%) [8]. Barriers to this therapy include, but are not limited to, lack of symptoms recognition and delayed presentation after symptoms onset, financial constraints, lack of drug and imaging modality availability (CTs), and provider related barriers (knowledge, experience, and fear of serious complications) [9, 10].

To date, there is no published outcome research regarding acute stroke care in Lebanon. Our study examined the utilization of thrombolytic therapy in patients presenting with acute stroke to a tertiary care center in Beirut, Lebanon. Patient characteristics and outcomes in addition to barriers to rt-PA utilization in this setting were also described comparing the two groups (rt-PA versus No rt-PA).

## 2. Methods

### 2.1. Study Setting and Design

The study was conducted in the Emergency department (ED) at the American University of Beirut Medical Center (AUBMC), the largest tertiary care center in Lebanon, with around 49,000 ED patient visits per year. The ED is staffed 38% of the time by American Board certified emergency medicine (EM) physicians and the rest of the time by physicians from different medical and surgical specialties. American Board certified neurologists and neurosurgeons are available for consultation 24 hours a day. Computer tomography (CT) imaging is available 24 hours of the day in the ED. The emergency medical services (EMS) agencies in Beirut are volunteer-based with Basic Life Support (BLS) level providers.

A retrospective chart analysis of adult patients who presented to the ED between June 1st 2009 and June 1st 2010 with symptoms of suspected stroke and who had a final hospital discharge diagnosis of ischemic cerebrovascular accident (CVA) was done. The study was approved by the AUB Institutional Review Board.

### 2.2. Inclusion/Exclusion Criteria

All adult patients (18 years and older) who presented to the ED during the study period with stroke symptoms were included if their corresponding chart had one of the following ICD-9 codes at hospital discharge: 434.00 (cerebral thrombosis without mention of cerebral infarction), 434.01 (cerebral thrombosis with cerebral infarction), 434.10 (cerebral embolism without mention of cerebral infarction), 434.11 (cerebral embolism with cerebral infarction), 434.90 (cerebral artery occlusion without mention of cerebral infarction), 434.91 (cerebral artery occlusion with cerebral infarction), 997.02 (iatrogenic cerebrovascular infarction or hemorrhage), E934.4 (adverse effects of fibrinolysis-affecting drugs), and 436 (acute but ill-defined cerebrovascular disease). Exclusion criteria were being of age <18 and missing ED charts. A total of 90 cases were reviewed. Three were excluded for missing charts. Eighty-seven patients were included in the study.

### 2.3. Data Collection

A data abstraction form was developed specifically for the purpose of this study. Charts were reviewed retrospectively and the data collected from the electronic health records included the following: patients' demographics and characteristics, ED presentation (signs and symptoms) information, radiology and laboratory results, time intervals (symptoms onset to ED presentation, symptoms onset to rt-PA administration), rt-PA utilization contraindications to rt-PA, hospital course (surgical procedures and MRI imaging), and patient outcomes (residual symptoms at hospital discharge, SICH, mortality). Modified Rankin scale (mRs) scores were also calculated for patients based on last neurologic exam prior to discharge from hospital. Patients with a mRs score of 2 or less were considered to have a favorable neurologic outcome. Patients with scores ≥3 were considered to have poor outcome at discharge [11].

### 2.4. Data Analysis

The Statistical Package for Social Sciences (SPSS), version 20.0, was used for data entry and analyses. Descriptive analyses were carried out by calculating the number and percent for categorical variables, whereas the mean, standard deviation (±SD), median and Interquartile Range (IQR) were calculated for continuous variables. In addition, Chi-square or Fisher's exact test was used to compare categorical variables between rt-PA and no rt-PA groups. The 2-sample *t*-test or the Mann-Whitney test was used to compare continuous variables between the two groups. A 95% confidence interval (CI) was calculated for overall rate of rt-PA administration. A *P* value of <0.05 was used to indicate statistical significance.

## 3. Results

A total of 87 patients were included in the study. These consisted of 50 males (57.5%) and 37 females (42.5%) with a mean age of 71.9 (SD = 11.8) years. Most patients arrived by private transport (85.1%). Weakness and loss of speech were the most common presenting signs (56.3%) followed by numbness (24.1%). Motor deficit was the most common physical finding (71.3%) followed by cranial nerve deficit (35.6%). The exact onset of symptoms was known in 82 patients (94.3%) with 48 patients (55.2%) presenting more than 6 hours after symptoms onset. Only 33 patients (37.9%) presented within 4.5 hours of symptom onset.

### 3.1. rt-PA Administration

Nine patients (10.3%, 95% CI (5.5–18.5)) received rt-PA: seven intravenous (IV) and two intra-arterial rt-PA. The mean time interval from symptom onset to IV rt-PA was 193.9 min (SD = 42.8). Door to IV rt-PA time (needle time) mean was 102.3 min (SD = 33.4). Delayed presentation (>6 hours) was the most common contraindication for rt-PA administration (55.2%) (Table 1).

### 3.2. rt-PA versus No rt-PA Groups Comparison for Patients Presenting within 4.5 Hours from Symptom Onset (Table 2)

Among patients presenting with acute stroke within the treatment window (<4.5 hours of symptom onset), eight patients received rt-PA (7 IV rt-PA and one intra-arterial rt-PA). An additional patient received intra-arterial rt-PA at 4.8 hours and was not included in the comparison presented in Table 2; both groups were similar in all other variables including demographics, past medical history, past surgical history, an medications including anticoagulation, presenting symptoms and signs, vital signs, and laboratory results (blood glucose, platelets, prothrombin time, international normalized ratio or INR). A trend of increased rt-PA use was found in cases staffed initially by EM physician when compared to another specialist (internal medicine or family medicine); however, this was not statistically significant (*P* = 0.24).

Among patients who presented within the treatment window (<4.5 hrs), those who received IV rt-PA had shorter mean door to CT completion time interval (49.4 ± 16.1 versus 190.3 ± 301.3 min *P* = 0.056).

### 3.3. Patient Outcomes

Most patients survived to hospital discharge (90.8%) with 15 having full resolution of their deficit. Five patients died during their hospital course (5.7%) and three had SICH (3.4%). There were no significant differences in complications (SICH and mortality) or outcomes (survival to hospital discharge, resolution of deficit, and mRs scores) when comparing patients in both groups (rt-PA or No rt-PA) (Table 3).

## 4. Discussion

The administration rate of rt-PA (10.3%) in our setting was higher than previously reported rates in other developing countries [8]. Despite the absence of a standardized stroke protocol in the ED at AUBMC, most of the recommendations that are adopted by stroke centers in developed countries to achieve good outcomes in acute stroke care [12] are available in our setting. These include “availability and interpretation of computed tomography scans 24 hours every day and rapid laboratory testing in addition to administrative support, strong leadership, and continuing education” [12]. Rapid neurology and neurosurgical team response is also available in our setting. The average door to IV rt-PA time interval of 102.4 min was however higher than the American Heart Association/American Stroke Association's recommended target of ≤60 min. The average door to CT completion time interval of 49.4 min is almost double the target time of 25 min. Process improvement in terms of faster triage of stroke patients, written care protocols, and the establishment of an acute stroke team would help reduce these time intervals [13]. More specifically, our institution is in the process of developing an IV rt-PA protocol. The implementation of such a protocol should streamline the care of stroke patients in the ED and improve door to CT and door to needle time [14]. A code stroke rapid protocol implemented by Nolte et al. not only resulted in reduction of median door to needle time but also contributed to an increase in rate of rt-PA use [15].

Delayed presentation was the main contraindication to rt-PA administration in our setting. More than half of the patients (55.2%) presented to ED more than 6 hours after the onset of symptoms. Although not directly addressed by our study, several factors including but not limited to poor recognition of stroke symptoms and failure to react appropriately in addition to failure to activate the EMS system (most of the patients used private transport instead of calling an ambulance) may have contributed to this delay in presentation. Studies examining barriers to rt-PA utilization in developed countries cite delayed presentation as the most important patient related barrier [16]. EMS use has been linked to reduced prehospital delays and increased likelihood of subsequent thrombolysis treatment [17]. Better patient education through public awareness campaigns is the main strategy recommended to address this barrier. The goal is to increase community knowledge regarding stroke risk factors, warning signs and symptoms and the need to act promptly since stroke is a time sensitive disease [17, 18]. Examining factors associated with prehospital delays in patients with acute stroke in our setting is also helpful to delineate focus areas for future initiatives aiming at reducing interval time from stroke onset to ED presentation. Factors associated with increased prehospital delays after symptom onset include increased age, worsening of symptoms, development of symptoms at home, and arrival at the ED by self or from other institutes [19–21].

On the other hand, factors that reduce the odds of delay in presentation after acute stroke include higher stroke severity and arrival by ambulance [19, 22]. In our study, a formal stroke scale such as National Institutes of Health Stroke Scale (NIHSS) was not used in the evaluation of patients for rt-PA administration. The adoption of a formal scale would improve characterizing better patients with acute stroke and allow for comparison with other settings.

### 4.1. Limitations

The results of our study should be considered in light of its limitations. First, the sample size was small which may have affected the power to detect a statistically significant difference in short-term outcomes in both groups of patients (rt-PA versus No rt-PA). Another limitation is inherent to the retrospective nature of the study; we did not examine long-term outcomes such as disability at 3 months where thrombolysis has been shown to have most impacts since this usually requires patient followup. A third is that our data was collected from only one medical center in Beirut. We assumed this sample would be representative of the overall urban population in Beirut. The rate of rt-PA utilization in other hospitals in Beirut is different since they vary in staffing, capabilities, and services provided for patients with acute stroke. Nonetheless, the study findings highlight important issues related to acute stroke treatment in our setting, which corroborates findings from similar studies in other developing countries.

## 5. Conclusion

In our setting, rt-PA utilization was higher than expected. Public education regarding stroke is needed to reduce preadmission barriers to rt-PA administration, decrease time from symptoms onset to ED presentation, and potentially improve outcomes further. A larger prospective study would help delineate more clearly other areas for possible interventions to improve outcomes in patients with acute stroke.

## Figures and Tables

**Table 1:** Time analysis for patients with IV rt-PA.

Symptoms onset to ED triage_*n* (%)	*N* = 87
0–4.5 hours	33 (37.9)
4.5–6 hours	6 (6.9)
>6 hours	48 (55.2)
Door to CT (min)*	*N* = 7
Mean (SD)	49.4 (16.1)
Median (IRQ)	51.0 (43.0–61.0)
Door to IV rt-PA (min)*	*N* = 7
Mean (SD)	102.4 (33.4)
Median (IRQ)	105.0 (80.0–110.0)
Symptoms onset to IV rt-PA (min)	*N* = 7
Mean (SD)	193.9 (42.8)
Median (IQR)	180.0 (150.0–240.0)

*patients who received IV rt-PA only.

**Table 2:** Characteristics of patients presenting within 4.5 hours from symptom onset by group (rt-PA versus no rt-PA).

Characteristics	All	No rt-PA	rt-PA	*P* value
Total sample	*N* = 33	*N* = 25	*N* = 8	
Gender				
Male	15 (45.5%)	12 (48.0%)	3 (37.5%)	0.70
Female	18 (54.5%)	13 (52.0%)	5 (62.5%)	
Age (mean, sd)	70.6 (13.6)	68.7 (14.3)	77.7 (6.9)	0.23
Arrival mode				
EMS	5 (15.2%)	5 (20.0%)	0 (0.0%)	0.30
Private	26 (78.8%)	19 (76.0%)	7 (87.5%)	
Unknown	2 (6.1%)	1 (4.0%)	1 (12.5%)	
Presenting symptoms				
Weakness	23 (69.7%)	18 (72.0%)	5 (62.5%)	0.67
Numbness	6 (18.2%)	6 (24.0%)	0 (0.0%)	0.30
Loss of vision	2 (6.1%)	1 (4.0%)	1 (12.5%)	0.43
Loss of speech	24 (72.7%)	17 (68.0%)	7 (87.5%)	0.39
Headache	33 (100.0%)	0 (0.0%)	0 (0.0%)	—
Dizziness	2 (6.1%)	2 (8.0%)	0 (0.0%)	1.00
Ataxia	1 (3.0%)	1 (4.0%)	0 (0.0%)	1.00
Decreased LOC	9 (27.3%)	9 (36.0%)	0 (0.0%)	0.07
Dysphagia/syncope/vertigo	0 (0.0%)	0 (0.0%)	0 (0.0%)	—
Fall	2 (6.1%)	2 (8.0%)	0 (0.05)	1.00
Past medical history				
HTN	28 (84.8%)	22 (88.0%)	6 (75.0%)	0.57
Diabetes	13 (39.4%)	10 (40.0%)	3 (37.5%)	1.00
CAD	9 (27.3%)	7 (28.0%)	2 (25.0%)	1.00
CHF	3 (9.1%)	3 (12.0%)	0 (0.0%)	0.56
AFIB/aflutter	7 (21.2%)	6 (24.0%)	1 (12.5%)	0.65
Dyslipidemia	16 (48.5%)	12 (48.0%)	4 (50.0%)	1.00
Peptic ulcer disease/GI bleed	0 (0.0%)	0 (0.0%)	0 (0.0%)	—
CVA	11 (33.3%)	10 (40.0%)	1 (12.5%)	0.22
Seizure	3 (9.1%)	2 (8.0%)	1 (12.5%)	1.00
TIA	3 (9.1%)	2 (8.0%)	1 (12.5%)	1.00
ICH	1 (3.0%)	1 (4.0%)	0 (0.0%)	1.00
Dementia	0 (0.0%)	0 (0.0%)	0 (0.0%)	—
Cancer	1 (3.0%)	0 (0.0%)	1 (12.5%)	0.24
Smoking				
Yes	10 (33.3%)	6 (27.3%)	4 (50.0%)	0.41
Previous	7 (23.3%)	5 (22.7%)	2 (25.0%)	
No	13 (43.3%)	11 (50.0%)	2 (25.0%)	
Medications				
Low molecular weight heparin	0 (0.0%)	0 (0.0%)	0 (0.0%)	—
Aspirin	16 (48.5%)	13 (52.0%)	3 (37.5%)	0.69
Clopidogrel	5 (15.6%)	4 (16.7%)	1 (12.5%)	1.00
Warfarin	5 (15.6%)	5 (20.8%)	0 (0.0%)	0.30
Physical examination				
Mental status change	7 (21.2%)	7 (28.0%)	0 (0.0%)	0.15
Motor deficit	25 (75.8%)	18 (72.0%)	7 (87.5%)	0.64
Sensory deficit	3 (9.4%)	3 (12.5%)	0 (0.0%)	0.56
Cranial nerve deficit	14 (42.4%)	9 (36.0%)	5 (62.5%)	0.24
Gait change	0 (0.0%)	0 (0.0%)	0 (0.0%)	—
Cerebellar deficit	2 (6.1%)	2 (8.0%)	0 (0.0%)	1.00
Babinski positive	12 (36.4%)	8 (32.0%)	4 (50.05)	0.42
Vital signs (mean sd)				
Systolic BP	153.0 (30.4)	152.3 (30.0)	156.2 (34.8)	0.69
Diastolic BP	78.0 (14.7)	76.4 (13.9)	84.8 (17.5)	0.23
Heart rate	83.0 (21.5)	82.7 (22.3)	84.5 (19.6)	0.83
Blood glucose	164.4 (82.3)	164.7 (83.0)	163.7 (87.3)	0.95
Laboratory (mean sd)				
Platelet count K	223.9 (64.9)	230.5 (68.4)	195.2 (38.8)	0.12
International normalized ratio (INR)	1.2 (0.4)	1.3 (0.4)	1.0 (0.0)	0.51
Prothrombin time (PT)	14.9 (5.0)	15.5 (5.3)	11.6 (0.1)	0.41
Partial thromboplastin time (PTT)	32.0 (7.1)	33.2 (7.5)	27.3 (1.8)	0.27
CT				
CT done	31 (93.9%)	23 (92.0%)	8 (100.0%)	1.00
Acute infarction	18 (54.5%)	16 (69.6%)	2 (25.0%)	0.04
MRI				
MRI done	24 (72.7%)	19 (76.0%)	5 (62.5%)	0.65
Acute infarction	21 (63.6%)	17 (89.5%)	4 (80.0%)	0.52
Physician specialty				
EM	18 (54.5%)	12 (48.0%)	6 (75.0%)	0.24
Other*	15 (45.5%)	13 (52.5%)	2 (25.0%)	

*Other (internal medicine and family medicine).

**Table 3:** Outcomes of patients by Group (rt-PA versus no rt-PA).

Outcome	No-rt-PA group	rt-PA group	*P* value
	*N* = 78	*N* = 9	
Complications			
None (%)	71 (91.0)	8 (88.9)	0.65
SICH (%)	3 (3.8)	0 (0)	1
Mortality (%)	4 (5.1)	1 (11.1)	0.5
Resolution of deficit at discharge (%)	14 (20.0)	1 (12.5)	1
Modified Rankin scale (mRs) score (%)			1
Favorable outcome (Score ≤2)	43 (58.1)	5 (55.6)	
Poor outcome (Score ≥3)	31 (41.9)	4 (44.4)	
Discharged alive (%)	74 (94.9)	8 (88.9)	0.65
